# CCR2 of Tumor Microenvironmental Cells Is a Relevant Modulator of Glioma Biology

**DOI:** 10.3390/cancers12071882

**Published:** 2020-07-13

**Authors:** Matthäus Felsenstein, Anne Blank, Alexander D. Bungert, Annett Mueller, Adnan Ghori, Irina Kremenetskaia, Olga Rung, Thomas Broggini, Kati Turkowski, Lea Scherschinski, Jonas Raggatz, Peter Vajkoczy, Susan Brandenburg

**Affiliations:** 1 Department of Experimental Neurosurgery Charité, Universitätsmedizin Berlin, Corporate Member of Freie Universität Berlin, Humboldt-Universität zu Berlin, and Berlin Institute of Health, 10117 Berlin, Germany; matthaeus.felsenstein@charite.de (M.F.); anne.blank@charite.de (A.B.); alexander.bungert@charite.de (A.D.B.); am2374@medschl.cam.ac.uk (A.M.); adnan.ghori@charite.de (A.G.); irina.kremenetskaia@charite.de (I.K.); olga.rung@web.de (O.R.); thomas.broggini@gmail.com (T.B.); kati.turkowski@mpi-bn.mpg.de (K.T.); lea.scherschinski@charite.de (L.S.); jonas.raggatz@outlook.com (J.R.); susan.brandenburg@charite.de (S.B.); 2 Department of Neurosurgery Charité, Universitätsmedizin Berlin, 10117 Berlin, Germany

**Keywords:** tumor-associated macrophages (TAMs), blood vessel integrity, tumor angiogenesis, CCR2/CCL2 signaling, GBM

## Abstract

Glioblastoma multiforme (GBM) shows a high influx of tumor-associated macrophages (TAMs). The CCR2/CCL2 pathway is considered a relevant signal for the recruitment of TAMs and has been suggested as a therapeutic target in malignant gliomas. We found that TAMs of human GBM specimens and of a syngeneic glioma model express CCR2 to varying extents. Using a *Ccr2*-deficient strain for glioma inoculation revealed a 30% reduction of TAMs intratumorally. This diminished immune cell infiltration occurred with augmented tumor volumes likely based on increased cell proliferation. Remaining TAMs in *Ccr2^-/-^* mice showed comparable surface marker expression patterns in comparison to wildtype mice, but expression levels of inflammatory transcription factors (*Stat3*, *Irf7*, *Cox2*) and cytokines (*Ifnβ*, *Il1β*, *Il12α*) were considerably affected. Furthermore, we demonstrated an impact on blood vessel integrity, while vascularization of tumors appeared similar between mouse strains. The higher stability and attenuated leakiness of the tumor vasculature imply improved sustenance of glioma tissue in *Ccr2^-/-^* mice. Additionally, despite TAMs residing in the perivascular niche in *Ccr2^-/-^* mice, their pro-angiogenic activity was reduced by the downregulation of *Vegf*. In conclusion, lacking CCR2 solely on tumor microenvironmental cells leads to enhanced tumor progression, whereby high numbers of TAMs infiltrate gliomas independently of the CCR2/CCL2 signal.

## 1. Introduction

Glioblastoma multiforme (GBM) is a poorly differentiated human brain tumor entity (WHO IV°) and displays markedly aggressive and invasive features. GBM tumors account for 60–70% of all malignant gliomas and have high recurrence rates with a dismal prognosis [1,2].

Large numbers of non-dysplastic cells belong to the glioma mass [3], including tumor-associated macrophages (TAMs) which make up 5–30% of all cells within the tumor compartment [4,5]. TAMs infiltrate due to chemotactic signals [6,7]. For example, CCL2 was shown to be released from glioma cells [5,8]. Its chemokine (C–C motif) receptor 2 (CCR2) is mainly located on the surface of TAMs [9,10]. The impact of this interaction has been demonstrated in several brain pathologies such as in atherosclerosis, autoimmune encephalitis, or even post-stroke inflammatory condition [11,12,13,14]. Furthermore, overexpression of CCL2 in glioblastoma rodent models showed that CCL2 increased the amount of infiltrating TAMs [15,16]. An anti-CCL2-antibody increased the survival time and reduced the number of TAMs [17]. Targeting the CCR2/CCL2 pathway showed effectiveness in other tumor models as it suppresses tumor progression [18,19,20]. Hence, the CCR2/CCL2 axis has been identified as an interesting target for tumor therapy, and collected data indicate that CCR2/CCL2 signaling plays a relevant role in the tumor-myeloid cell-interaction. Yet, the precise function of CCR2-signaling for glioma progression as well as angiogenesis has not been elucidated. 

Previously, the function of CCR2/CCL2 signal for glioma biology was investigated by focusing on its ligand CCL2. Both, *Ccl2^-/-^* mice and anti-CCL2 antibodies were investigated [17,21,22]. In our study, we used *Ccr2^-/-^* mice [23] in combination with the GL261 immunocompetent mouse glioma model to evaluate the importance of the CCR2 expression on the tumor microenvironment, especially on TAM recruitment and their phenotype as well as glioma vascularization. We demonstrate an impaired influx of myeloid cells in *Ccr2*-deficient animals, whereby remaining TAMs displayed a varied expression of pro-inflammatory molecules. Ultimately, we observed enhanced tumor growth and an increase in tumor blood vessel integrity. Using the *Ccr2^-/-^* knockout model, severe glioma microenvironmental alterations were uncovered, which do not appear beneficial.

## 2. Results

### 2.1. CCR2-Expression of Microglia/Macrophages in Human and Murine Glioblastoma Tissue

**Figure 1:**
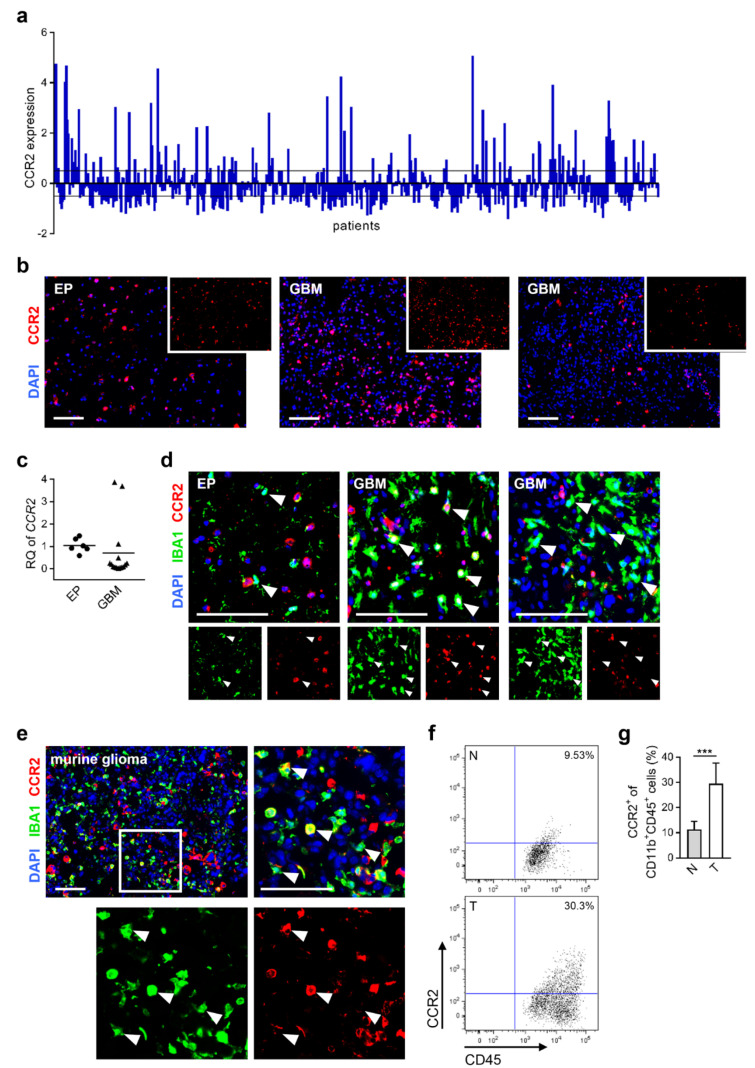
CCR2 is expressed within human and murine glioma tissues. (**a**) Expression data of *CCR2* from glioblastoma patients were used from TCGA Affymetrix U133A data (528 GBM patient samples). The relative gene expression of each patient is depicted (each column represents one patient). Black lines define the z-score threshold (0.5). (**b**) Human epilepsy (EP) and GBM brain tissue sections stained for CCR2. Scale bars 100 µm (n = 3–6). (**c**) Myeloid cells (CD11b^+^) were isolated from freshly homogenized human brain tissues, and RNA was extracted. Realtime-PCR for *CCR2* is presented (n = 6–15). (**d**) Representative images of frozen human brain sections stained for IBA1 and CCR2 are depicted (arrowheads define IBA1^+^ cells). Scale bars 100 µm (n = 3–6). (**e**) Murine brain tumor sections were analyzed for IBA1 and CCR2 on day 21 of glioma growth. A representative image of the intratumoral area is shown (square illustrates magnification of tumor tissue area; *arrowheads* define IBA1^+^ cells expressing CCR2). Scale bars 100 µm (n = 3). (**f**) Murine brain cells were stained with CD11b, CD45, and CCR2 antibodies and analyzed via flow cytometry. Dot plots represent the expression of CCR2 of CD11b^+^CD45^+^ myeloid cells within naïve (N) and tumor-bearing (T) brains. (**g**) Graph depicts percentage of CCR2^+^ cells within the CD11b^+^CD45^+^ cell fraction (n = 10–12). *** *p* < 0.001 (unpaired Student’s *t*-test).

It has been demonstrated that CCR2/CCL2 signaling plays a pivotal role in chemo-attraction during neuro-inflammatory processes [15,24]. Analyzing the expression level of *CCR2* within GBM patient samples using the TCGA database, we found that 51.5% of all patient specimens show regulated *CCR2* expression (19.3% up-regulation and 32.2% down-regulation; Figure 1a). Additionally, we investigated surgically resected human brain tissues at our institution and observed CCR2 positive staining both in epilepsy (EP) (3/3) as well as GBM tissue samples (6/6) revealing variable expression levels in glioma specimens (Figure 1b). Moreover, we detected *CCR2* expression in freshly isolated myeloid cells (CD11b^+^) derived from corresponding human brain specimens. CD11b^+^ cells of GBM samples predominantly displayed down-regulation with respect to myeloid cells from epilepsy tissues, but two samples showed strong up-regulation of *CCR2* in GBM (Figure 1c). Additionally, we detected CCR2 positive TAMs in three of six human GBM samples after immunofluorescence staining while no CCR2 protein was expressed in IBA1^+^ cells of EP tissues (Figure 1d). This suggests the relevance of the CCR2/CCL2 signaling for myeloid cells in a subset of human GBMs. To find a suitable model system, we investigated CCR2 expression in mice with BL6/J background. We observed CCR2 expression in TAMs of tumors of the syngeneic GL261 glioma mouse model both on RNA [25] and protein level (Figure 1e). Flow cytometric analyses revealed only minor expression of CCR2 on myeloid cells (CD11b^+^CD45^+^) in naïve mouse brains while CCR2 was up-regulated in tumor-bearing mice (Figure 1f). The percentage of CCR2^+^ cells within the CD11b^+^CD45^+^ myeloid cell population increased significantly (Figure 1g) implicating their reactivity on the CCR2/CCL2 signaling pathway.

Altogether, we found a remarkable expression of CCR2 in tumor-associated macrophages in human and murine glioblastomas. To understand the role of CCR2 within the tumor microenvironment, we used a suitable rodent tumor model in combination with *Ccr2^-/-^* mice.

### 2.2. Diminished Infiltration of TAMs in Brain Tumor Tissues of Ccr2KO Mice

**Figure 2:**
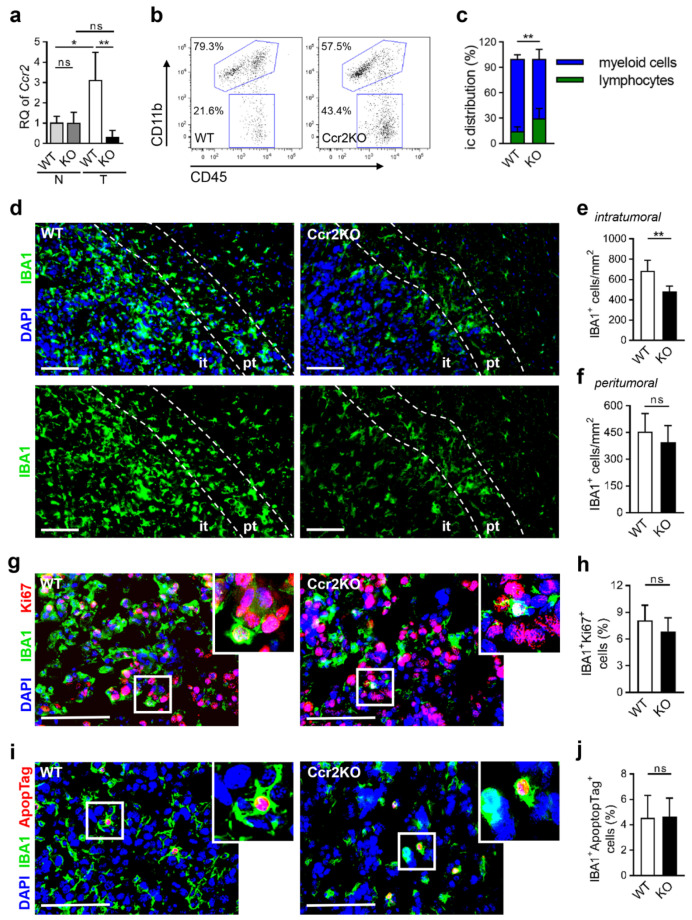
IBA1^+^ cells accumulate less in tumor-bearing *Ccr2*-deficient mouse brains. (**a**) RT-PCR for *Ccr2* of CD11b^+^ cells from naïve (N) and tumor-bearing (T; d21) brain tissues of the wildtype (WT), as well as Ccr2KO (KO) mice, is presented (n = 3–4). ** *p* < 0.01; * *p* < 0.05; ns, not significant (one-way ANOVA and Bonferroni’s multiple comparison test). (**b**) Tumor-bearing brains of WT and Ccr2KO mice (d21) were analyzed by flow cytometry. Representative dot plots show gates for TAMs (CD11b^+^CD45^+^) and lymphocytes (CC11b^-^CD45^+^). (**c**) The graph depicts the calculation of myeloid cells and lymphocytes (n = 10–11). (**d**) Tumors of WT and Ccr2KO brains were analyzed via immunofluorescence staining for myeloid cells (IBA1^+^; d21). Representative images of the tumor border are depicted. *Dashed lines* define the peritumoral area (pt; 100 µm region from tumor border into normal brain tissues) and surround the intratumoral area (it). Scale bars 100 µm. (**e**,**f**) The calculation for the number of IBA1^+^ cells within the it (e) and pt (f) area are illustrated (n = 6). (**g**,**i**) Representative images of immunofluorescence staining for IBA1 and Ki67 (g) or apoptosis (i) in the tumor area of WT and Ccr2KO mice (d21). *Squares* illustrate magnified areas. Scale bars 100 µm. (**h**,**j**) Graphs depict rate of proliferation (h) and calculation of apoptotic IBA1^+^ cells (j) (n = 6). ** *p* < 0.01; ns, not significant (unpaired Student’s *t*-test).

TAMs were isolated from naïve and tumor-bearing wildtype (WT) as well as *Ccr2^-/-^* (Ccr2KO) mice. RNA has been extracted with subsequent gene expression analyses. As expected, Ccr2KO mice did not express *Ccr2* following 21-days of glioma growth, in strong contrast with WT animals (Figure 2a). We sought to investigate homogenized brain tumor tissues for immune cell infiltration and observed a remarkable shift (Figure 2b). In Ccr2KO mice, the myeloid cell fraction (CD11b^+^CD45^+^) was significantly reduced while the lymphocyte population (CD11b^-^CD45^+^) increased (Figure 2c). However, total numbers of T cells, whether effector T cells or immune-suppressive regulatory T cells, were unchanged (Figure S1a–c), and B cell count was slightly reduced (Figure S1d). The decrease in TAMs was verified by immunofluorescence staining (Figure 2d). In particular, intratumoral areas showed reduced numbers of TAMs in *Ccr2*-deficient mice (Figure 2e) while peritumoral regions were not affected (Figure 2f). We suggest that the altered ratio of myeloid cells and lymphocytes was likely based on diminished TAM influx. To exclude basal differences in myeloid cell distribution in the CNS, microglia of naïve mice were counted revealing similar numbers and comparable morphology in wildtype and transgenic mice (Figure S2a,b). In gliomas, both microglia and macrophages can infiltrate the tumor tissue while their individual contribution is intensely debated [7,26]. Even if we did not analyze them separately in vivo, we stimulated microglia from naïve brain tissues and also generated macrophages from bone marrow with tumor-conditioned media. We observed that stimulated microglia and macrophages of both mouse strains expressed equal markers in vitro, e.g., IBA1, CD11b, CD68, and F4/80, respectively (Figure S2c–e) demonstrating viable and inducible myeloid cells independently of *Ccr2*. *Ccr2*-deficiency is known to affect the migration of TAMs [23], however, their proliferative and apoptotic activity may be additionally influenced, which could explain differences in TAM counts of the intratumoral area. We, therefore, stained for Ki67 (Figure 2g) and DNA fragmentation (Figure 2i) of IBA1^+^ cells but could not detect changes in proliferation (Figure 2h) or apoptosis (Figure 2j).

In line with the current literature, we assume that the reduced accumulation of TAMs in glioma tissue of Ccr2KO mice is predominantly due to diminished CCR2-dependent migration rather than alteration of their cellular activity. However, 70% of myeloid cells infiltrate the tumor tissue independently of the CCR2/CCL2 signaling axis (Figure 2e).

### 2.3. Ccr2-Deficiency Leads to Accelerated Brain Tumor Growth

**Figure 3:**
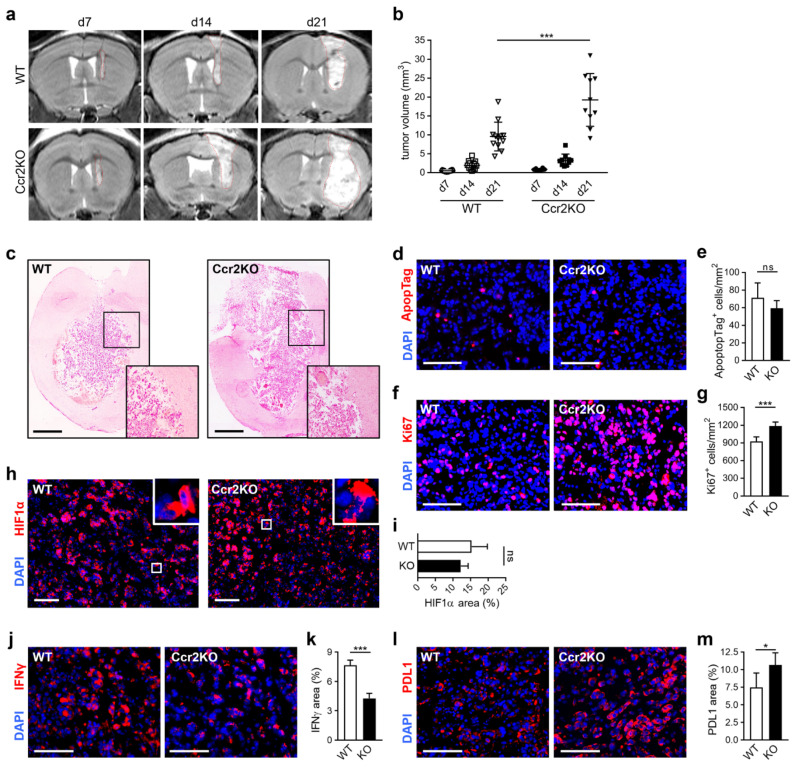
Glioma growth is enhanced in *Ccr2*-deficient mice. (**a**) Tumor sizes of gliomas were measured with MRI on day 7, 14, and 21 after inoculation with GL261 cells. Representative MRI images are shown for the WT (upper row) and Ccr2KO (lower row) mice. Red dashed lines define tumor borders. (**b**) Graph displays calculated tumor volumes (n = 10–12). *** *p* < 0.001 (one-way ANOVA and Bonferroni’s multiple comparison test). (**c**) Representative images of hematoxylin-eosin-stained tissue sections of tumor-bearing WT and Ccr2KO brains are shown for day 21 (n = 4–5). *Squares* indicate magnified regions of the tumor border. Scale bars 1000 µm. (**d**,**f**) Apoptosis (d) and proliferation (f) of the intratumoral area (d21) were analyzed by immunofluorescence staining of ApopTag (d) and Ki67 (f). Scale bars 100 µm. (**e**,**g**) Graphs depict the amount of apoptotic (**e**) and proliferative (**g**) cells within glioma tissues (n = 6). (**h**) HIF1α was stained to detect hypoxia in tumor tissues (d21). Squares indicate magnified regions. Scale bars 100 µm. (**i**) Calculation for area of HIF1α expression (n = 5). (**j**–**m**) Tissues were stained for IFNγ (j) and PDL1 (l). Scale bars 100 µm. Positive areas of IFNγ (k) and PDL1 were calculated (m) (n = 4–5). *** *p* < 0.001; * *p* < 0.05; ns, not significant (unpaired Student’s t-test).

In other tumor models, it has been described that *Ccr2*-deficiency led to reduced tumor sizes [20]. Hence, we analyzed glioma progression at consecutive time points using MRI (Figure 3a). Unexpectedly, we measured an increase of tumor volumes at day 7 and 14, while sizes even doubled in Ccr2KO mice compared to WT at day 21 (Figure 3b). Early animal loss in the Ccr2KO group up to day 21 has not been observed. However, survival experiments were not performed. Despite augmented tumor progression, no differences in glioma cell invasion were noticed after the histopathological examination (Figure 3c). Moreover, we assessed the cellular activity of tumor tissues and discovered no differences in apoptosis (Figure 3d,e) but an increased cell proliferation in Ccr2KO animals (Figure 3f) by up to 25% (Figure 3g). The rapid and uncontrolled tumor cell proliferation restricts the ability of oxygen. Therefore, hypoxia is a typical feature of gliomas [27,28]. Here, we analyzed hypoxia-regulated molecules such as HIF1α and BNIP3 that are commonly up-regulated during tumor progression [27,28,29]. HIF1α, as a pivotal hallmark in response to hypoxia, showed strong expression (Figure 3h), but no differences between mouse strains were observed (Figure 3i). In our glioma model, we found BNIP3 located in the nuclei (Figure S3a). This localization is associated with increased tumor cell survival [29,30]. But again, WT and Ccr2KO mice revealed comparable results (Figure S3b). Thus, despite the higher proliferative activity of tumors in Ccr2KO mice, we could not detect significant changes in hypoxic area distribution implicating a sufficient supply of oxygen and nutrients in these tumors. In addition, we investigated the expression of immune relevant molecules that can modulate the tumor microenvironment. We focused on interferon-gamma (IFNγ), a cytokine which facilitates antitumor immunity [31] and programmed death ligand 1 (PDL1) relevant for mediation of immunosuppression [32]. Interestingly, we found reduced IFNγ expression (Figure 3j,k) and increased PDL1 signal (Figure 3l,m) in gliomas of *Ccr2^-/-^* mice that indicated a rather immunosuppressive milieu in these tumors.

Our results demonstrate that the reduced number of infiltrating TAMs is accompanied by accelerated glioma growth, an increased tumor cell proliferation, and a more tumor supportive micromilieu in Ccr2KO mice. 

### 2.4. TAMs of Ccr2KO Mice Show Classical Markers but Exhibit a Modified Expression of Inflammatory Genes

**Figure 4:**
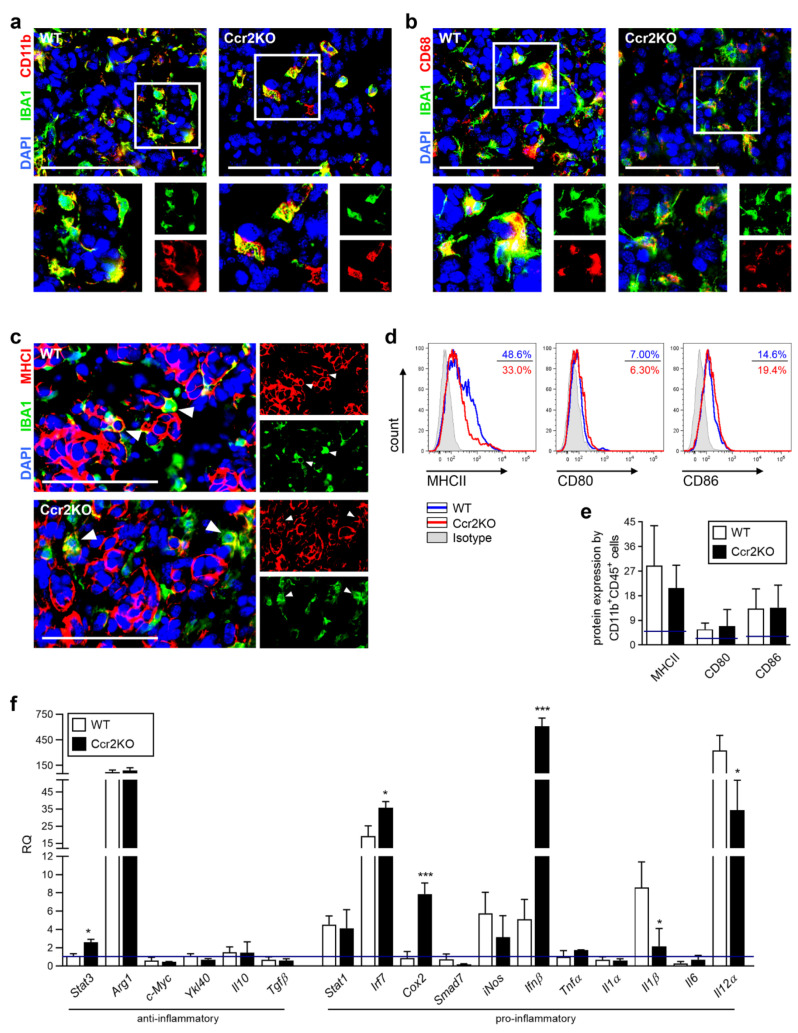
Tumor-associated macrophages (TAMs) of WT and Ccr2KO mice carry similar surface markers but show differences in expression of transcription factors as well as cytokines at day 21 of tumor growth. (**a**–**c**) Representative images of brain tumor sections of WT and Ccr2KO mice stained for IBA1 and CD11b (a) or CD68 (b) or MHCI (c) are depicted (n = 4–5). Squares indicate magnified regions (a,b). Arrowheads define IBA1^+^ cells expressing MHCI (c). Scale bars 100 µm. (**d**) Brain tumor suspensions were analyzed by flow cytometry after staining with CD11b, CD45, and MHCII, CD80, or CD86 antibodies. Histograms represent molecule expression of CD11b^+^CD45^+^ myeloid cells within WT and Ccr2KO mice including isotype controls. (**e**) The graph depicts the percentage of molecule expression (n = 6–14). *Blue lines* define basic molecule expression in naïve mice. (**f**) RT-PCRs for indicated genes of CD11b^+^ cells from tumor-bearing brain tissues of WT as well as Ccr2KO mice are presented (n = 3–4). *Blue line* defines basic molecule expression in naïve WT mice. *** *p* < 0.001; * *p* < 0.05 (unpaired Student’s *t*-test).

Despite enormous research efforts to understand the TAM function in glioma, their precise role remains unclear. Recent studies identified these cells as a mixed population with pro-inflammatory and immune suppressive properties [14,33], but ultimately promote tumor growth [25]. Consequently, TAMs were likely involved in the tumor progression of recently applied glioma models. To assess the general functionality of TAMs, we validated class-identifying molecules. Characteristic surface markers of tumor-associated macrophages, like CD11b and CD68, were found in gliomas of both mouse strains, without noticeable differences in expression (Figure 4a,b). Furthermore, we investigated antigen-presenting molecules (MHCI, MHCII) as well as costimulatory ligands (CD80, CD86) important for the induction of immune cell responses. As previously defined, only low amounts of TAMs carry MHCI in murine glioma tissues [34] which we were also able to detect in Ccr2KO mice (Figure 4c). The expression of MHCII and co-stimulatory molecules of Ccr2KO were comparable to WT (Figure 4d,e). However, analyzing the expression of various immune-regulatory genes, belonging to the family of transcription factors or cytokines, revealed changes of TAMs in glioma-bearing hemispheres of Ccr2KO mice (Figure 4f) while the basic expression of some genes of microglia from naïve mice already vary (Table S2). We identified *Stat3*, *Irf7*, *Cox2,* and *Ifnβ* as being up-regulated, while *Il1β* and *Il12α* show down-regulation under tumor condition. *Ifnβ*, that displayed strong up-regulation, has previously been described to induce expression of PDL1 in a subpopulation of myeloid cells, the myeloid-derived suppressor cells (MDSCs) [35] which are known to have immune-suppressive functions. Thus, we investigated the expression of PDL1 by IBA1^+^ cells within the tumor area. We found a slight increase of TAMs that express PDL1 but without reaching significance (Figure S4).

Thus, myeloid cells accumulating in intratumoral areas of Ccr2KO mice are characterized by similar expression of TAM-markers and surface molecules required for T cell activation. However, transcription factors as well as soluble molecules were expressed differently without meaningful polarization of the myeloid cells in *Ccr2*-deficient mice. 

### 2.5. Improved Vascular Integrity in Ccr2-Deficient Mice

**Figure 5:**
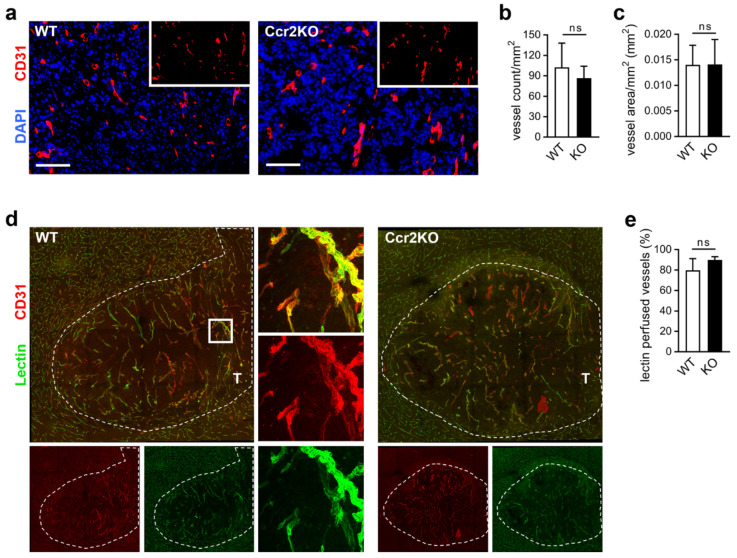
Glioma vasculature is unaffected by *Ccr2*-deficiency. (**a**) Representative images of brain tumor sections of WT and Ccr2KO mice stained for CD31 are depicted (d21). Scale bars 100 µm. (**b,c**) Graphs indicate vessel count (b) and vascularized area (c) within the tumor tissues (n = 6). (**d**) Confocal microscopy of brain tumor sections from mice perfused with FITC-Lectin and stained for CD31 (overview, mosaic). *Square* indicates the magnified region (63×). *T*, tumor tissue; *dashed line*, tumor border. (**e**) The graph presents the percentage of perfused vessels (n = 3–4). ns, not significant (unpaired Student’s *t*-test).

Sustained angiogenesis is regarded as a central hallmark of cancer development, and stimulation of blood vessel growth leads to the mandatory supply of nutrients and oxygen during glioma growth [16]. Changes in vascularization may be responsible for increased tumor sizes. Therefore, we analyzed the vascular structure by immunofluorescence staining. To exclude basal differences, the contralateral hemisphere of WT and Ccr2KO brains were investigated (Figure S5a), revealing comparable vessel densities (Figure S5b) and vascularized areas (Figure S3c). Comparing tumor tissue of both mouse strains (Figure 5a), we found similar vessel counts (Figure 5b) and no changes in the blood vessel covered areas (Figure 5c). Additionally, the vasculature of the peritumoral area appeared to be unaffected by *Ccr2*-deficiency (Figure S5d–f). Overall, a similar vascular structure of both mouse strains has been characterized.

**Figure 6:**
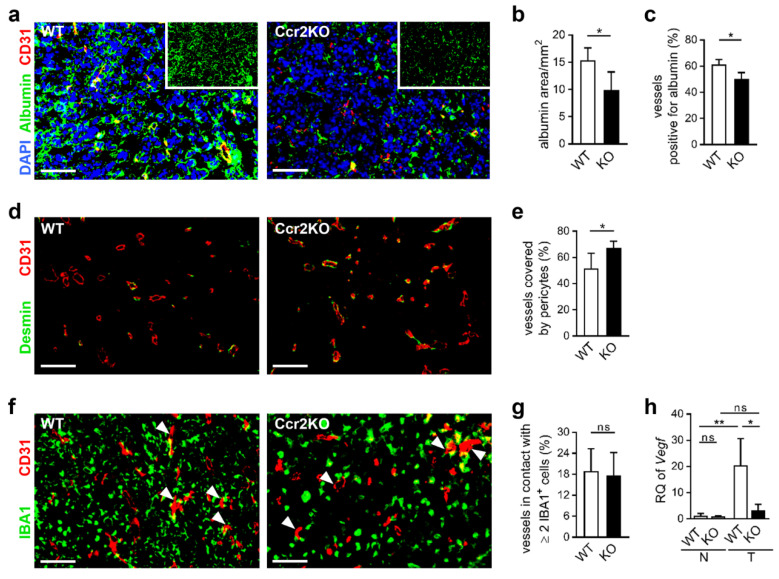
Vascular integrity is improved of glioma grown in *Ccr2*-deficient mice. (**a,d,f**) Representative images of brain tumor sections of WT and Ccr2KO mice (d21) stained for CD31 and albumin (a), Desmin (d) or IBA1 (f) are depicted. Arrowheads indicate vessels in contact with IBA1^+^ cells (f). Scale bars 100 µm. (**b,c,e,g**) Graphs present area of albumin staining (b), vessels colocalized with albumin (c), pericyte-covered vessels (e) and vessels interacting with IBA1^+^ cells (g). * *p* < 0.05; ns, not significant (unpaired Student’s *t*-test). (**h**) RT-PCR for *Vegf* of CD11b^+^ cells (myeloid cells) from naïve (N) and tumor-bearing (T) brain tissues of WT as well as Ccr2KO mice is depicted (d21; n = 3–4). ** *p* < 0.01; * *p* < 0.05; ns, not significant (one-way ANOVA and Bonferroni´s multiple comparison test).

Still, tumor blood vessels can be characterized by increased permeability and leakiness, leading to impaired function [36]. To study the integrity of blood vessels in tumor mice we analyzed vessel perfusion and stability. Injection of FITC-labeled lectin (Figure 5d) revealed comparable fractions of lectin perfused tumor vessels in WT and Ccr2KO mice (Figure 5e). Interestingly, the albumin covered area was reduced in glioma tissues of Ccr2KO animals (Figure 6a,b) accompanied by less albumin-stained tumor vessels (Figure 6c) indicating increased stability and less permeability of blood vessels dependent on *Ccr2*-deficiency. In addition, vessels of tumors in Ccr2KO mice showed a greater coverage with pericytes (Figure 6d,e), which can also be a sign for higher maturity of blood vessels [37]. We recently demonstrated that TAMs are often associated with glioma vasculature and involved in angiogenesis by expression of pro-angiogenic factors [25]. Interestingly, despite the reduction of TAM counts in transgenic mice, the interaction of IBA1^+^ cells with tumor blood vessels remained at approximately 18% (Figure 6f,g). This indicates a preference of TAMs for the perivascular niche in Ccr2KO mice, which could facilitate the improved vessel functionality. Therefore, we investigated whether TAMs of Ccr2KO mice are involved in pericyte recruitment or angiogenesis by expression of various genes such as *Pdgfβ*, *Ang1/2*, and *Mmp2/9*, but were not able to discover significant alterations compared to the wildtype (Figure S5g). However, *Ccr2*-deficiency caused a reduction of the angiogenic molecule *Vegf* in TAMs of the tumor-bearing hemisphere (Figure 6h), likely due to improved nutrient and oxygen supply of stabilized vessels. 

Thus, we observed enhanced vascular integrity in tumors of Ccr2KO mice, which may be one of the responsible factors for augmented tumor growth.

## 3. Discussion

Overall, we investigated the impact of *Ccr2*-deficiency in the glioma microenvironment on tumor progression. We have been able to show variable CCR2 expression in human GBM tissues and high expression in murine gliomas. Using the syngeneic GL261 glioma model, increased tumor volumes in *Ccr2^-/-^* mice were measured, accompanied by reduced infiltration of TAMs and higher integrity of blood vessels.

The decrease in myeloid cell recruitment has previously been observed in *Ccr2*-knockout tumor models [18,20]. However, analyzing the impact of *Ccr2*-deficiency on tumor sizes and vascularization led to different results. In prostate cancer, tumor volumes were unaffected, while cell proliferation and angiogenesis were diminished [18]. In lung carcinoma, smaller tumors with strong vascular remodeling were found [20]. Thus, the relevance of CCR2 on tumor progression varies and depends on the tumor’s environmental context and location. The glioma microenvironment in the immune-privileged brain is different due to resident microglia that accumulate in addition to peripheral macrophages [5]. A recent study described only a small impact on the survival of glioma-bearing *Ccr2* transgenic mice whereby tumor volumes were not evaluated [38]. Due to our experimental set-up with a focus on glioma biology and a defined endpoint, we were not able to link our results to survival data of this study. Nevertheless, the authors observed reduced numbers of TAMs with immunosuppressive features as well [38]. Targeting the ligand of CCR2, CCL2, in prostate cancer [18,19] and glioma [17,21] led to a reduction of tumor growth and prolonged survival. Under these conditions, not only the recruitment of TAMs is inhibited but also the attraction of regulatory T cells via CCR4 [22] and the autocrine mechanism on glioma cells, expressing CCL2 and CCR2 [39], is interrupted [18,40]. Thus, observations in our used knockout mouse model could be referred to diminished TAM infiltration, while targeting CCL2 led to additional inhibitory effects of the CCR2/CCL2 pathway.

Following up on our experiments, *Ccr2*-knockout impaired the intratumoral TAM accumulation but was not entirely impeded. Still, 70% of IBA1^+^ cells were recruited into the glioma area independently of the CCR2/CCL2 signal. Considering current literature, it is assumed that only macrophages express CCR2 [21,22]. This would imply, preferential infiltration of microglia into gliomas of *Ccr2^-/-^* mice because these immune cells can migrate independently of CCR2. However, final evidence for this hypothesis is lacking, and we were not differentiating between these both cell populations. Even if CCL2 is believed to be the major recruiter of TAMs in gliomas [15,16,41,42], other factors like CX3CL1 [43], CXCL12, M-CSF and GM-CSF [44] can mobilize TAMs into brain tumor areas, and become relevant under *Ccr2*-deficiency. Besides IBA1^+^ cells, we also investigated the infiltration of effector T cells as well as regulatory T cells but detected no significant differences while B cell counts were slightly reduced. A more differential classification of T and B lymphocytes and their distribution was not performed, further challenging a detailed characterization of the immune cell composition in *Ccr2*^-/-^ mice.

Remaining intratumoral TAMs in *Ccr2^-/-^* mice appeared functional and showed classical features of the monocytic lineage, characterized by carrying IBA1, CD68, CD11b as well as antigen-presenting molecules. Isolated CD11b^+^ cells from tumors of both mouse strains expressed anti- and pro-inflammatory molecules, revealing a mixed phenotype as expected for the wildtype [33]. We referred to this cell population from tumor-bearing mice to myeloid cells. This cell population could be composed of microglia and macrophages which carry similar surface markers [26], while neutrophils could be precluded in this glioma model [34]. We decided not to further stratify subpopulations based on lacking evidence of their specific functions. It should be noted that we observed partly different basal expression of genes in microglia of naïve wildtype and *Ccr2^-/-^* animals. We assume that the detected differences in gene expression of myeloid cells during glioma progression are relevant, but at this point, we are not able to pre-exclude an influence of divergent baseline values.

The molecular signature of TAMs did not display a specific tumor-supportive polarization nor appear to induce the production of immunosuppressive cytokines *Il10* or *Tgfβ.* Nevertheless, we detected a reduction of markers like *iNos*, *Il1β,* and *Il12α,* important for tumor growth inhibition [43,45], when comparing *Ccr2*-deficient TAMs to wildtype cells. Furthermore, CD11b^+^ cells of Ccr2KO mice overexpressed *Ifnβ* and *Stat3*, molecules that are relevant for the immunosuppressive phenotype of MDSCs [35]. However, we could not find elevated PDL1 expression within the TAMs of *Ccr2*-deficient mice that should be induced by *Ifnβ* [35]. Even if PDL1 is unchanged in the IBA1 cell population, we observed an overall increase of PDL1 expression in the tumor tissue of *Ccr2^-/-^* mice. Notably, tumor cells can utilize the PD1/PDL1 pathway to circumvent the immune surveillance by expression of PDL1 [32], and in human glioma, PDL1 was found to be a negative prognostic indicator [46]. While PDL1 is expressed by tumor cells and immune cells, IFNγ is primarily expressed by T cells as well as NK cells, and characterized as an anti-proliferative agent [31]. We did not co-stain IFNγ with immune cell markers. Nevertheless, we found reduced IFNγ positive staining in the glioma area. These data implicate an attenuated anti-tumor response and a rather immunosuppressive tumor micromilieu in *Ccr2*-deficient mice which could support the enhanced glioma progression.

Nutritional vessel supply and tumor-induced angiogenesis are important malignant characteristics of the GBM and are regarded as pivotal hallmarks of carcinogenesis [16]. Here, the blood-brain barrier integrity is frequently lost due to the angiogenic activity of the tumor [47], which allows for passive transport of cells and proteins as well as the reorganization of the vascular structure. We found higher pericyte coverage of tumor blood vessels and less albumin extravasation. Pericytes retain blood-brain barrier function and are known to be involved in vessel stability [37]. Our data implicate reduced blood-brain barrier degradation in *Ccr2*-deficient mice, which has previously been demonstrated in other brain diseases [48]. We speculate that the tightened blood-brain barrier supports the decreased infiltration of myeloid cells from the periphery and encourages tumor maintenance.

Our study aimed to elucidate the role of *Ccr2*-deficiency within the tumor microenvironment. Here, we focused on the myeloid cell population due to their well-characterized CCR2 expression [9,10], but also other leukocytes and endothelial cells express CCR2 under respective conditions [49,50]. Indeed, we found CCR2 positive cells negative for IBA1 in human and murine tumor tissues. We speculate that these are mainly glioma cells that are known to express CCR2 [39]. However, tumor microenvironmental cells other than the myeloid cells were not analyzed in detail. Thus, we cannot pre-exclude their contribution to the observed effects in the glioma mouse model used. In contrast with previous studies, which targeted the CCR2/CCL2 pathway primarily in alternative experimental models [40,51], we found enhanced tumor volumes in *Ccr2^-/-^* mice. However, divergent data obtained in transgenic mouse models and under treatment conditions have been described previously [52]. It should be considered that targeting antibodies ubiquitously affect all receptors or ligands, respectively. In comparison, by using the model applied here, only cells of the tumor microenvironment (e.g., myeloid cells, endothelial cells) possess *Ccr2*-deficiency, while glioma cells still express *Ccr2*, hence retaining functionality towards CCL2 and other signals. We assume that if the entire CCR2/CCL2 signaling axis is inflicted by antibody treatment, tumor growth is inhibited, while specific knockout of tumor microenvironmental cells by using a *Ccr2^-/-^* transgenic mice leads to enhanced glioma progression. This progression is accompanied by reduced TAM accumulation, the strong proliferation of tumor cells, sufficient oxygen, and nutrient supply of the tumor tissue, ultimately supported by stabilized vasculature in *Ccr2^-/-^* mice. We speculate that CCR2-dependent myeloid cells are crucial to controlling glioma growth while the remaining CCR2-independent TAMs are unable to arrest or decelerate tumor expansion. We demonstrate that a more detailed investigation of TAMs is required to identify and characterize tumor-controlling subpopulations, which likely helps to find more effective treatment options.

## 4. Materials and Methods 

### 4.1. Human Specimens

Tissue samples were obtained during therapeutic surgical treatment (Department of Neurosurgery, Charité Universitätsmedizin Berlin, Germany) from 2013 to 2014. Here, epilepsy patients (6 cases) who underwent temporal pole resection, and patients suffered from GBM (15 cases) were included. Neuropathologists assessed WHO tumor grade by standard prognostic markers. 

All procedures involving human specimens were carried out following the ethical standards of the national research committee and consistent with the Helsinki Declaration and its later amendments or comparable ethical standards. Approval of the Ethical Committee of Charité-Universitätsmedizin Berlin was received (application number: EA4/065/13) and all analyses were carried out following the defined obligations of scientific working with patient material. Informed consent was obtained from all subjects.

Tissue samples were embedded in 4% PFA for 24 h and subsequently dehydrated in a serial dilution of sucrose. Afterward, samples were frozen in liquid nitrogen. Frozen sections of 10 µm were prepared. Another tissue part was used for microglia/macrophage isolation.

Gene expression data were achieved from the GBM patient dataset available through The Cancer Genome Atlas (TCGA; Affymetrix U133A [53,54]).

### 4.2. Animals

C57Bl6/J mice (WT) were obtained from Charles River Laboratories (Sulzfeld, Germany). Homozygous *Ccr2^-/-^* animals (C57Bl6/J background; [23,55]) were received from Prof. Dr. Mathias Heikenwälder (HMGU Munich and DKFZ Heidelberg, Germany) and bred at FEM Bayer. Experiments were performed with sex- and age-matched animals. All animal experiments were conducted according to German Laws for Animal Protection and the National Institute of Health Guidelines for Care and Use of Laboratory Animals (LaGeSo No. G0152/09; G0281/14).

### 4.3. Intracerebral Tumor Cell Inoculation

The tumor cell line GL261 was cultivated in high-glucose Dulbecco´s modified Eagles medium (DMEM) Gibco™ (ThermoFisher Scientific, Waltham, MA, USA) and contained stable glutamine and sodium pyruvate, 10% fetal calf serum, 100 units of penicillin/mL and 100 μg/mL of streptomycin. Tumor cells were incubated for three days at 37 °C and 5% CO_2_ atmosphere until they reached a confluence of 80%. Cells were diluted in PBS. 2 × 10^4^ cells/μL were used for stereotactic cell inoculation. Anesthetized mice were fixed in a stereotactic frame. The position for stereotactic implantation was 1 mm anterior and 2 mm lateral to the right from bregma. Via Hamilton syringe (Roth, Karlsruhe, Germany) 1 μL cell solution was injected into parenchyma at 3 mm depth.

### 4.4. MRI Analysis

Animals were measured with a 7.0 Tesla animal scanner (Bruker Pharma Scan^®^, Billerica, MA, USA). To achieve high contrast and resolution, contrast agent Magnevist^®^ (Bayer AG, Leverkusen, Germany) was injected into the tail vein. Mice were anesthetized with 1.5–2.0% isoflurane (Forene, Abbot, Abbot Park, IL, USA) in a narcotic mixture, together with O2/N2O (30/70%). Brain slices were recorded with T1- and T2-weighted 2-dimensional turbo spin-echo sequences by Paravision 4.0 software (Bruker Bio Spin^®^, Ettlingen, Germany). Volumes of tumors were calculated with Analyze 5.0 (AnalyzeDirect, Overland Park, KS, USA).

### 4.5. Lectin Application

Mice were anesthetized, and 0.1 mL of 1 mg/mL lectin (Dylight488 Lycopersicon Esulentum (Tomato) Lectin; Vector Laboratories, Burlingame, CA, USA) was slowly injected via tail vein. After 5 min, mouse was perfused with 4% paraformaldehyde (PFA, Sigma-Aldrich, St. Louis, MO, USA).

### 4.6. Cardial Brain Perfusion

To prepare tissue for histological analysis, at day 21 animals were anesthetized. PBS or PFA were used for perfusion into the left heart-ventricle. Brains were directly used for the preparation of brain cell suspensions or post-fixed in 4%-PFA and dehydrated in ascending sucrose solution (10%/20%/30%). Frozen brains were processed in a microtome. 10 μm or 50 µm sections were prepared and stored at −80 °C.

### 4.7. Immunohistochemistry

For hematoxylin-eosin-staining, frozen sections were fixed in absolute ethanol, dyed with hematoxylin and subsequently rinsed with water. Afterwards, sections were dyed in eosin, followed by a short wash in tap water. Then, ascending ethanol series and finally, xylene was used for tissue dehydration. The Zeiss Axio Observer Z1 Microscope (Zeiss MicroImaging GmbH, Jena, Germany) was used for taking mosaic pictures.

### 4.8. Immunofluorescence Staining

Brain sections were blocked with PBS/0.5% Casein (Sigma-Aldrich) for 30 min. The following primary antibodies were used: rb anti-IBA1 (1:250; Wako Chemicals, Neuss, Germany), gt anti-IBA1 (1:100; Abcam, Cambridge, UK), rat anti-CD31 (1:50; BD Pharmingen, Heidelberg, Germany), rat anti-CD4 (1:50; BD Pharmingen), rat anti-CD8a (1:50; BD Pharmingen), rat anti-CD45R (B220; 1:50; BD Pharmingen), rb anti-Desmin (1:100; Abcam), rb anti-Albumin, rat anti-CD11b (1:100; Abcam), rat anti-CD68 (1:200; AbDSerotec, Oxfordshire, UK), rat anti-MHCI (1:100; Abcam), rb anti-Ki67 (1:100; ThermoFisher Scientific), gt anti-HIF1α (1:50; R&D Systems), rb anti-BNIP3 (1:100; Abcam), rb anti-PDL1 (1:100; Abcam), rb anti-IFNγ (1:100; Abcam), rb anti-FOXP3 (1:50; Abcam), gt anti-mouse CCR2 (1:100; Abcam), mouse anti-human CCR2 (1:50; Abcam). After 2 h incubation, sections were washed two times with PBS/0.5% Casein. Secondary antibodies were applied to sections: anti-rb Dylight488, anti-rb AF488, anti-gt Dylight488, anti-rat FITC, anti-rat Cy3, anti-rb Cy3, anti-gt Cy3, anti-mouse Rhodamine Red, anti-rb AF647 or anti-gt AF647 (1:200; Dianova, Hamburg, Germany). Tissue was subsequently incubated for 1.5 h. Sections were washed with PBS and water. Finally, slices were covered with DAPI-containing mounting medium (Dianova). 

For staining of Desmin, before blocking step, sections were fixed 10 min at −20 °C with Methanol, and for detection of CCR2 on mouse sections Antigen Retrieval Reagent (R&D Systems, Minneapolis, MN, USA) was used for 30 s. For staining of HIF1α, BNIP3, PDL1, IFNγ, and FOXP3, sections were permeabilized by 0.1% Triton X-100 (Sigma-Aldrich) in PBS or TBS (FOXP3) for 10 min. The staining of FOXP3 was performed by using TBS instead of PBS in all steps of the procedure.

For detection of apoptotic cells, sections were stained with Apoptosis Detection Kit (ApopTag^®^, Merck Millipore, Burlington, MA, USA), based on TUNEL-technique, according to manufacturer protocol. Finally, IBA1-staining was performed as described. 

Human brain sections were treated with Autofluorescence Eliminator Reagent (Millipore) accordingly manufacturer’s instructions before blocking.

Images were acquired by fluorescence microscope Zeiss Axio Observer Z1 (Zeiss MicroImaging GmbH) or confocal microscope SP8 (Leica Biosystems, Nussloch, Germany).

Sections were analyzed systematically. For each tumor, 3 to 8 sections were investigated and up to 9 images per section were taken depending on tumor size and staining. Images were analyzed by ImageJ Software (NIH; https://imagej.nih.gov/ij/).

### 4.9. Cell Homogenization

On day 21, mice were perfused with 10 mL PBS, brains extracted and cerebella, as well as olfactory bulbs, removed. Brains of mice or 400 mg human brain tissue were minced and digested in enzyme mixtures from the Papain Neuronal Tissue Dissociation Kit (Miltenyi Biotec, Bergisch Gladbach, Germany) according to the manufacturer´s protocol. Cell suspensions were filtered (Pre-separation filter, 30 µm, Miltenyi Biotec). Homogenates were used either for direct flow cytometric analyses or specific CD11b^+^ cell isolation.

### 4.10. Isolation of CD11b^+^ Cells

Following mechanic and enzymatic digestion of mouse and human brain tissues, cells were labeled with anti-CD11b magnetic microbeads (Miltenyi Biotec). The cell suspension was added to a MACS LS column (Miltenyi Biotec), which was placed in a magnetic field. Non-labeled cells were washed out, and CD11b positive cells were eluted. This procedure was repeated with an MS column (Miltenyi Biotec). Positive cells with high purity (>95%) were achieved.

### 4.11. Isolation of Bone Marrow Cells and Generation of Macrophages

Femurs of WT and *Ccr2^-/-^* mice were rinsed with PBS. Following washing, the cells were plated into a cell culture flask with RPMI medium 1640 (ThermoFisher Scientific; including 10% FCS, 1% penicillin/streptomycin) and 20 ng/mL recombinant M-CSF (Miltenyi Biotec). The medium was changed every second day for eight days. Cells were detached by a cell scraper and used for culture experiments. Macrophages with a purity of 90% were obtained.

### 4.12. RNA Isolation Real-Time PCRs

The isolated CD11b^+^ cells of murine and human brains were used for RNA extraction via PureLink™ RNA Mini Kit (Invitrogen™, Carlsbad, CA, USA) according to the manufacturer’s instructions. Here, CD11b^+^ cells of six murine brain tumor hemispheres or three naïve brains were pooled to generate one RNA sample. The quality of RNA was verified with Agilent 2100 Bioanalyzer (Agilent Technologies, Santa Clara, CA, USA). Reverse transcription was performed with purified RNA using QuantiTect Reverse Transcription Kit (Qiagen, Hilden, Germany) according to the manufacturer instructions. For semi-quantitative real-time PCR, reactions were performed in triplicates in a volume of 25 µL, containing 12.5 µL of SYBR Premix Ex Taq Kit (Takara Bio Inc., Kusatsu, Japan), forward and reverse primers, following the manufacturer’s protocol. Used primers are listed in Supplementary Table S1. Expression levels were normalized to 18S mRNA levels. Relative expression levels were determined by the ^ΔΔ^Ct method.

### 4.13. Culture of Myeloid Cells In Vitro and Immunocytochemistry

For the cultivation of microglia following isolation by anti-CD11b microbeads from naïve mouse brains, cells were seeded at a density of 2 × 10^5^ cells/well in 8 well glass bottom chamber slides (ThermoFisher Scientific) and cultured in tumor-conditioned DMEM medium for seven days. The medium was changed every second day.

For the cultivation of macrophages, cells were seeded at a density of 1.2 × 10^5^ cells/well in 8 well glass bottom chamber (ThermoFisher Scientific) and cultured in tumor-conditioned RPMI medium 1640 for four days. The medium was changed every second day.

The tumor-conditioned medium was obtained by collecting the supernatant of GL261 cells which were grown for three days to 80% confluence in DMEM or RPMI 1640. The supernatant was centrifuged two times by maximum speed to exclude glioma cells. 

Immunocytochemistry of microglia and macrophages was performed after fixation of the cells with 4% paraformaldehyde in PBS for 20 min. Subsequently, microglia were permeabilized for 10 min with 0.3% Triton X-100 in PBS and blocked in 1% Casein/PBS for 1 h. Primary antibodies were incubated 2 h at room temperature: rat anti-CD11b (1:100; Abcam), rat anti-CD68 (1:100; AbDSerotec), and rabbit anti-IBA1 (1:200; WAKO). For visualization, cells were incubated with fluorochrome-conjugated secondary antibodies (anti-rabbit Dylight488 and anti-rat Cy3; Dianova) for 1.5 h protected from light. The macrophages were treated for 10 min with methanol at −20 °C and blocked in 1% Casein/PBS for 30 min. Primary antibodies were incubated 2 h at room temperature: rat anti-F4/80 (1:100; Abcam) and goat anti-IBA1 (1:100; Abcam). As secondary antibodies, anti-rat Cy3 and anti-goat AF647 (Dianova) were used. After staining, chambers were removed and slides were mounted with DAPI-containing mounting medium (Dianova). Images were acquired at 20× magnification using an inverse fluorescence microscope Zeiss Axio Observer Z1 (Carl Zeiss MicroImaging GmbH).

### 4.14. Flow Cytometry

To assess immune cells, cells were washed with PBS/0.5% BSA. Staining was performed with PE anti-CD11b (BD Pharmingen) and APC anti-CD45 (BD Pharmingen), or FITC anti-CD11b (1:100; BD Pharmingen), PE anti-CCR2 (1:20; R&D Systems) and APC anti-CD45 (1:200). Isotype control rat IgG2b (R&D Systems) was used for CCR2-antibody. Subsequently, cells were incubated for 20 min on ice. DAPI was added to detect dead cells. 

For other surface molecules, cells were fixed. Here, the cell suspension was washed with PBS, fixed for 20 min with 2% PFA, and washed with PBS/0.5% BSA. Cells were permeabilized with 0.5% saponin (exception MHCII, PBS/0.5% BSA) and staining was performed in 0.5% saponin (MHCII only 0.5% BSA/PBS). Antibodies PE anti-CD11b (1:200) and APC anti-CD45 (1:200) were used in combination with the respective specific antibodies coupled to FITC: anti-I-Ab (1:50), anti-CD80 (1:100), anti-CD86 (1:100) as well as isotype controls rat IgG2a, Armenian hamster IgG and mouse IgG2a (BioLegend, San Diego, CA, USA). Cells were incubated for 20 min at room temperature. Cells were washed with 0.5% saponin and analyzed by flow cytometry. 

All samples were analyzed with BD FACS Canto II (BD Pharmingen) and evaluated with FlowJo software (Ashland, OR, USA).

### 4.15. Statistical Analysis

All data presented with mean and error bars indicate standard deviation. GraphPad Prism Software (San Diego, CA, USA) was used to calculate statistical significance. Differences were evaluated by two-tailed unpaired Student´s t-test or as indicated. Significant values were presented as *p* < 0.05.

## 5. Conclusions

In our study of a murine glioma model, the reduced accumulation of TAMs in *Ccr2*-deficient mice is accompanied by increased tumor volumes based on the enhanced proliferation of glioma cells, changes in their inflammatory gene expression and improved integrity of tumor blood vessels. Notably, approximately 70% of TAMs infiltrated glioma tissues independently of the CCR2/CCL2 signal which underlines the role of other important recruitment factors.

## Supplementary Materials

The following are available online at https://www.mdpi.com/2072-6694/12/7/1882/s1, Figure S1: Infiltration of T cells into tumor tissues is unaffected by *Ccr2*-deficiency but B cell counts are reduced, Figure S2: Myeloid cells of naïve *Ccr2*-deficient mice show same morphology and marker expression as the wildtypes, Figure S3: BNIP3 is localized in the nucleus and expressed by tumors of the wildtype and Ccr2KO mice, Figure S4: Vascular structure of brains is similar between mouse strains. Table S1: Primer sequences, Table S2: Basic expression of molecules.

## References

[1] Louis D.N., Ohgaki H., Wiestler O.D., Cavenee W.K., Burger P.C., Jouvet A., Scheithauer B.W., Kleihues P. (2007). The 2007 WHO classification of tumours of the central nervous system. Acta Neuropathol..

[2] Ohgaki H., Kleihues P. (2005). Epidemiology and etiology of gliomas. Acta Neuropathol..

[3] Charles N.A., Holland E.C., Gilbertson R., Glass R., Kettenmann H. (2011). The brain tumor microenvironment. Glia.

[4] Roggendorf W., Strupp S., Paulus W. (1996). Distribution and characterization of microglia/macrophages in human brain tumors. Acta Neuropathol..

[5] Watters J.J., Schartner J.M., Badie B. (2005). Microglia function in brain tumors. J. Neurosci. Res..

[6] Kushchayev S.V., Kushchayeva Y.S., Wiener P.C., Scheck A.C., Badie B., Preul M.C. (2014). Monocyte-derived cells of the brain and malignant gliomas: The double face of Janus. World Neurosurg..

[7] Müller A., Brandenburg S., Turkowski K., Müller S., Vajkoczy P. (2015). Resident microglia, and not peripheral macrophages, are the main source of brain tumor mononuclear cells. Int. J. Cancer.

[8] Badie B., Schartner J., Klaver J., Vorpahl J. (1999). In vitro modulation of microglia motility by glioma cells is mediated by hepatocyte growth factor/scatter factor. Neurosurgery.

[9] Zhai H., Heppner F.L., Tsirka S.E. (2011). Microglia/macrophages promote glioma progression. Glia.

[10] Ellert-Miklaszewska A., Dabrowski M., Lipko M., Sliwa M., Maleszewska M., Kaminska B. (2013). Molecular definition of the pro-tumorigenic phenotype of glioma-activated microglia. Glia.

[11] Komohara Y., Horlad H., Ohnishi K., Fujiwara Y., Bai B., Nakagawa T., Suzu S., Nakamura H., Kuratsu J., Takeya M. (2012). Importance of direct macrophage-tumor cell interaction on progression of human glioma. Cancer Sci..

[12] Markovic D.S., Vinnakota K., Chirasani S., Synowitz M., Raguet H., Stock K., Sliwa M., Lehmann S., Kalin R., van Rooijen N. (2009). Gliomas induce and exploit microglial MT1-MMP expression for tumor expansion. Proc. Natl. Acad. Sci. USA.

[13] Galarneau H., Villeneuve J., Gowing G., Julien J.P., Vallieres L. (2007). Increased glioma growth in mice depleted of macrophages. Cancer Res..

[14] Umemura N., Saio M., Suwa T., Kitoh Y., Bai J., Nonaka K., Ouyang G.F., Okada M., Balazs M., Adany R. (2008). Tumor-infiltrating myeloid-derived suppressor cells are pleiotropic-inflamed monocytes/macrophages that bear M1- and M2-type characteristics. J. Leukoc. Biol..

[15] Platten M., Kretz A., Naumann U., Aulwurm S., Egashira K., Isenmann S., Weller M. (2003). Monocyte chemoattractant protein-1 increases microglial infiltration and aggressiveness of gliomas. Ann. Neurol..

[16] Zhang J., Sarkar S., Cua R., Zhou Y., Hader W., Yong V.W. (2012). A dialog between glioma and microglia that promotes tumor invasiveness through the CCL2/CCR2/interleukin-6 axis. Carcinogenesis.

[17] Zhu X., Fujita M., Snyder L.A., Okada H. (2011). Systemic delivery of neutralizing antibody targeting CCL2 for glioma therapy. J. Neurooncol..

[18] Izhak L., Wildbaum G., Jung S., Stein A., Shaked Y., Karin N. (2012). Dissecting the autocrine and paracrine roles of the CCR2-CCL2 axis in tumor survival and angiogenesis. PLoS ONE.

[19] Sanford D.E., Belt B.A., Panni R.Z., Mayer A., Deshpande A.D., Carpenter D., Mitchem J.B., Plambeck-Suess S.M., Worley L.A., Goetz B.D. (2013). Inflammatory monocyte mobilization decreases patient survival in pancreatic cancer: A role for targeting the CCL2/CCR2 axis. Clin. Cancer Res..

[20] Schmall A., Al-Tamari H.M., Herold S., Kampschulte M., Weigert A., Wietelmann A., Vipotnik N., Grimminger F., Seeger W., Pullamsetti S.S. (2015). Macrophage and cancer cell cross-talk via CCR2 and CX3CR1 is a fundamental mechanism driving lung cancer. Am. J. Respir. Crit. Care Med..

[21] Chen Z., Feng X., Herting C.J., Garcia V.A., Nie K., Pong W.W., Rasmussen R., Dwivedi B., Seby S., Wolf S.A. (2017). Cellular and Molecular Identity of Tumor-Associated Macrophages in Glioblastoma. Cancer Res..

[22] Chang A.L., Miska J., Wainwright D.A., Dey M., Rivetta C.V., Yu D., Kanojia D., Pituch K.C., Qiao J., Pytel P. (2016). CCL2 Produced by the Glioma Microenvironment Is Essential for the Recruitment of Regulatory T Cells and Myeloid-Derived Suppressor Cells. Cancer Res..

[23] Kuziel W.A., Morgan S.J., Dawson T.C., Griffin S., Smithies O., Ley K., Maeda N. (1997). Severe reduction in leukocyte adhesion and monocyte extravasation in mice deficient in CC chemokine receptor 2. Proc. Natl. Acad. Sci. USA.

[24] Prinz M., Mildner A. (2011). Microglia in the CNS: Immigrants from another world. Glia.

[25] Brandenburg S., Muller A., Turkowski K., Radev Y.T., Rot S., Schmidt C., Bungert A.D., Acker G., Schorr A., Hippe A. (2016). Resident microglia rather than peripheral macrophages promote vascularization in brain tumors and are source of alternative pro-angiogenic factors. Acta Neuropathol..

[26] Glass R., Synowitz M. (2014). CNS macrophages and peripheral myeloid cells in brain tumours. Acta Neuropathol..

[27] Muz B., de la Puente P., Azab F., Azab A.K. (2015). The role of hypoxia in cancer progression, angiogenesis, metastasis, and resistance to therapy. Hypoxia (Auckl).

[28] Jensen R.L. (2009). Brain tumor hypoxia: Tumorigenesis, angiogenesis, imaging, pseudoprogression, and as a therapeutic target. J. Neurooncol..

[29] Burton T.R., Henson E.S., Baijal P., Eisenstat D.D., Gibson S.B. (2006). The pro-cell death Bcl-2 family member, BNIP3, is localized to the nucleus of human glial cells: Implications for glioblastoma multiforme tumor cell survival under hypoxia. Int. J. Cancer.

[30] Burton T.R., Eisenstat D.D., Gibson S.B. (2009). BNIP3 (Bcl-2 19 kDa interacting protein) acts as transcriptional repressor of apoptosis-inducing factor expression preventing cell death in human malignant gliomas. J. Neurosci..

[31] Castro F., Cardoso A.P., Goncalves R.M., Serre K., Oliveira M.J. (2018). Interferon-Gamma at the Crossroads of Tumor Immune Surveillance or Evasion. Front. Immunol..

[32] Alsaab H.O., Sau S., Alzhrani R., Tatiparti K., Bhise K., Kashaw S.K., Iyer A.K. (2017). PD-1 and PD-L1 Checkpoint Signaling Inhibition for Cancer Immunotherapy: Mechanism, Combinations, and Clinical Outcome. Front. Pharmacol..

[33] Szulzewsky F., Pelz A., Feng X., Synowitz M., Markovic D., Langmann T., Holtman I.R., Wang X., Eggen B.J., Boddeke H.W. (2015). Glioma-associated microglia/macrophages display an expression profile different from M1 and M2 polarization and highly express Gpnmb and Spp1. PLoS ONE.

[34] Brandenburg S., Turkowski K., Mueller A., Radev Y.T., Seidlitz S., Vajkoczy P. (2017). Myeloid cells expressing high level of CD45 are associated with a distinct activated phenotype in glioma. Immunol. Res..

[35] Xiao W., Klement J.D., Lu C., Ibrahim M.L., Liu K. (2018). IFNAR1 Controls Autocrine Type I IFN Regulation of PD-L1 Expression in Myeloid-Derived Suppressor Cells. J. Immunol..

[36] Farnsworth R.H., Lackmann M., Achen M.G., Stacker S.A. (2014). Vascular remodeling in cancer. Oncogene.

[37] Holm A., Heumann T., Augustin H.G. (2018). Microvascular Mural Cell Organotypic Heterogeneity and Functional Plasticity. Trends Cell Biol..

[38] Flores-Toro J.A., Luo D., Gopinath A., Sarkisian M.R., Campbell J.J., Charo I.F., Singh R., Schall T.J., Datta M., Jain R.K. (2020). CCR2 inhibition reduces tumor myeloid cells and unmasks a checkpoint inhibitor effect to slow progression of resistant murine gliomas. Proc. Natl. Acad. Sci. USA.

[39] Liang Y., Bollen A.W., Gupta N. (2008). CC chemokine receptor-2A is frequently overexpressed in glioblastoma. J. Neurooncol..

[40] Loberg R.D., Day L.L., Harwood J., Ying C., St John L.N., Giles R., Neeley C.K., Pienta K.J. (2006). CCL2 is a potent regulator of prostate cancer cell migration and proliferation. Neoplasia.

[41] Leung S.Y., Wong M.P., Chung L.P., Chan A.S., Yuen S.T. (1997). Monocyte chemoattractant protein-1 expression and macrophage infiltration in gliomas. Acta Neuropathol..

[42] Desbaillets I., Tada M., de Tribolet N., Diserens A.C., Hamou M.F., Van Meir E.G. (1994). Human astrocytomas and glioblastomas express monocyte chemoattractant protein-1 (MCP-1) in vivo and in vitro. Int. J. Cancer.

[43] Wu S.Y., Watabe K. (2017). The roles of microglia/macrophages in tumor progression of brain cancer and metastatic disease. Front. Biosci. (Landmark Ed.).

[44] Roesch S., Rapp C., Dettling S., Herold-Mende C. (2018). When Immune Cells Turn Bad-Tumor-Associated Microglia/Macrophages in Glioma. Int. J. Mol. Sci..

[45] Mantovani A., Sozzani S., Locati M., Allavena P., Sica A. (2002). Macrophage polarization: Tumor-associated macrophages as a paradigm for polarized M2 mononuclear phagocytes. Trends Immunol..

[46] Nduom E.K., Wei J., Yaghi N.K., Huang N., Kong L.Y., Gabrusiewicz K., Ling X., Zhou S., Ivan C., Chen J.Q. (2016). PD-L1 expression and prognostic impact in glioblastoma. Neuro. Oncol..

[47] Engelhardt B., Liebner S. (2014). Novel insights into the development and maintenance of the blood-brain barrier. Cell Tissue Res..

[48] Varvel N.H., Neher J.J., Bosch A., Wang W., Ransohoff R.M., Miller R.J., Dingledine R. (2016). Infiltrating monocytes promote brain inflammation and exacerbate neuronal damage after status epilepticus. Proc. Natl. Acad. Sci. USA.

[49] Salcedo R., Ponce M.L., Young H.A., Wasserman K., Ward J.M., Kleinman H.K., Oppenheim J.J., Murphy W.J. (2000). Human endothelial cells express CCR2 and respond to MCP-1: Direct role of MCP-1 in angiogenesis and tumor progression. Blood.

[50] Fujimura N., Xu B., Dalman J., Deng H., Aoyama K., Dalman R.L. (2015). CCR2 inhibition sequesters multiple subsets of leukocytes in the bone marrow. Sci. Rep..

[51] Teng K.Y., Han J., Zhang X., Hsu S.H., He S., Wani N.A., Barajas J.M., Snyder L.A., Frankel W.L., Caligiuri M.A. (2017). Blocking the CCL2-CCR2 Axis Using CCL2-Neutralizing Antibody Is an Effective Therapy for Hepatocellular Cancer in a Mouse Model. Mol. Cancer Ther..

[52] Winnicka B., O’Conor C., Schacke W., Vernier K., Grant C.L., Fenteany F.H., Pereira F.E., Liang B., Kaur A., Zhao R. (2010). CD13 is dispensable for normal hematopoiesis and myeloid cell functions in the mouse. J. Leukoc. Biol..

[53] Cerami E., Gao J., Dogrusoz U., Gross B.E., Sumer S.O., Aksoy B.A., Jacobsen A., Byrne C.J., Heuer M.L., Larsson E. (2012). The cBio Cancer Genomics Portal: An Open Platform for Exploring Multidimensional Cancer Genomics Data. Cancer Discov..

[54] Gao J., Aksoy B.A., Dogrusoz U., Dresdner G., Gross B., Sumer S.O., Sun Y., Jacobsen A., Sinha R., Larsson E. (2013). Integrative analysis of complex cancer genomics and clinical profiles using the cBioPortal. Sci Signal..

[55] Boring L., Gosling J., Chensue S.W., Kunkel S.L., Farese R.V., Broxmeyer H.E., Charo I.F. (1997). Impaired monocyte migration and reduced type 1 (Th1) cytokine responses in C-C chemokine receptor 2 knockout mice. J. Clin. Investig..

