# Supplementary material for: CCR2 of Tumor Microenvironmental Cells Is a Relevant Modulator of Glioma Biology
Source: Cancers (Basel). 2020 Jul 13;12(7):1882. doi: 10.3390/cancers12071882 (PMC7408933; doi:10.3390/cancers12071882)
Supplement: Supplementary file 1 [file cancers-12-01882-s001.zip › MF Supplementary Figures.pdf]

CCR2 of tumor microenvironmental cells is a relevant modulator of glioma biology

Matthäus Felsenstein [1,‡], Anne Blank [1], Alexander D. Bungert [1], Annett Mueller [1], Adnan Ghori [1], Irina Kremenetskaia [1], Olga Rung [1], Thomas Broggini [1,‡], Kati Turkowski [1], Lea Scherschinski [1], Jonas Raggatz [1], Peter Vajkoczy [1,2,†,*] and Susan Brandenburg [1,†]

Supplementary Figures

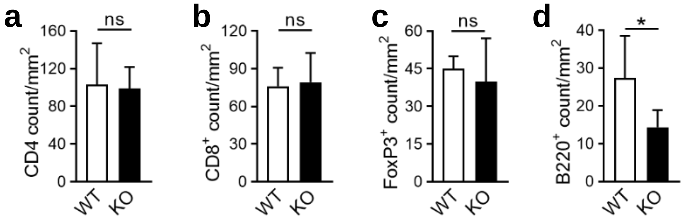

Figure S1. Infiltration of T cells into tumor tissues is unaffected by *Ccr2*-deficiency but B cell counts are reduced. Brain tumor tissues of WT and Ccr2KO mice (d21) were stained for CD4, CD8, FoxP3 and B220 (n= 3-5). Graphs depict numbers of counted T helper cells ($CD4^+$) **(a)**, cytotoxic T cells ($CD8^+$) **(b),** regulatory T cells ($FoxP3^+$) **(c)** and B lymphocytes ($B220^+$) **(d)**. ns, not significant; *$p < 0.05$ (unpaired Student's t test).

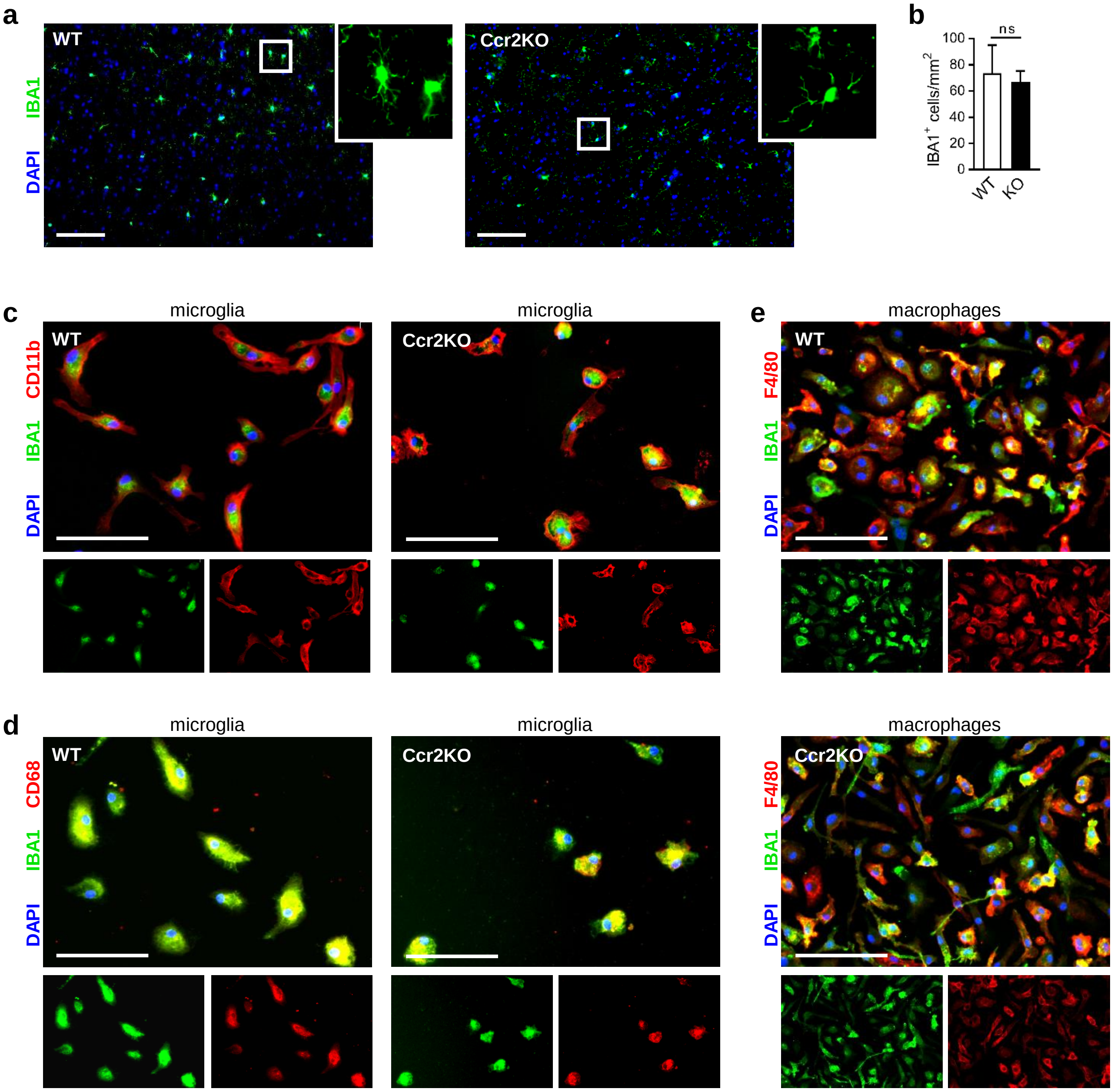

Figure S2. Myeloid cells of naïve *Ccr2*-deficiant mice show same morphology and marker expression as the wildtypes. **(a)** Representative images of immunofluorescence staining for IBA1 of contralateral hemispheres of WT and Ccr2KO mouse brains. *Squares* illustrate magnified areas. *Scale bars* 100 µm. **(b)** Graph depicts IBA1^{+} cell count on brain sections from non-tumor bearing hemispheres (n= 5-6). ns, not significant (unpaired Student's t test). **(c,d)** Myeloid cells were isolated from naïve WT and Ccr2KO brains using the surface molecule CD11b for selection. Isolated cells were cultured in tumor-conditioned medium for seven days. Cells were stained for IBA1 and CD11b **(c)** and CD68 **(d)**. *Scale bars* 100 µm. **(e)** Isolated bone marrow cells of WT and Ccr2KO mice were cultured with M-CSF for eight days. Afterwards macrophages were harvested and seeded in tumor-conditioned medium for four days. Cells were stained for IBA1 and F4/80. *Scale bars* 100 µm.

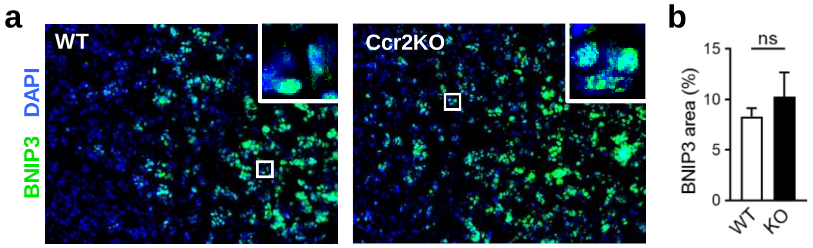

Figure S3. BNIP3 is localized in the nucleus and expressed by tumors of the wildtype and Ccr2KO mice. **(a)** BNIP3 was stained on tumor-bearing brains (d21). *Squares* indicate magnified regions. 20x magnification. **(b)** Graph depicts area of BNIP3 expression (n= 5). ns, not significant (unpaired Student's t test).

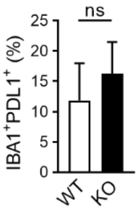

Figure S4. Percentage of TAMs expressing PDL1 is comparable between wildtype and Ccr2KO mice. PDL1 is stained with IBA1 on tumor-bearing brain sections of wildtype and *Ccr2*$^{-/-}$ mice (d21). Graph depicts calculation of IBA1^{+} cells expressing PDL1 (n= 4-5). ns, not significant (unpaired Student's t test).

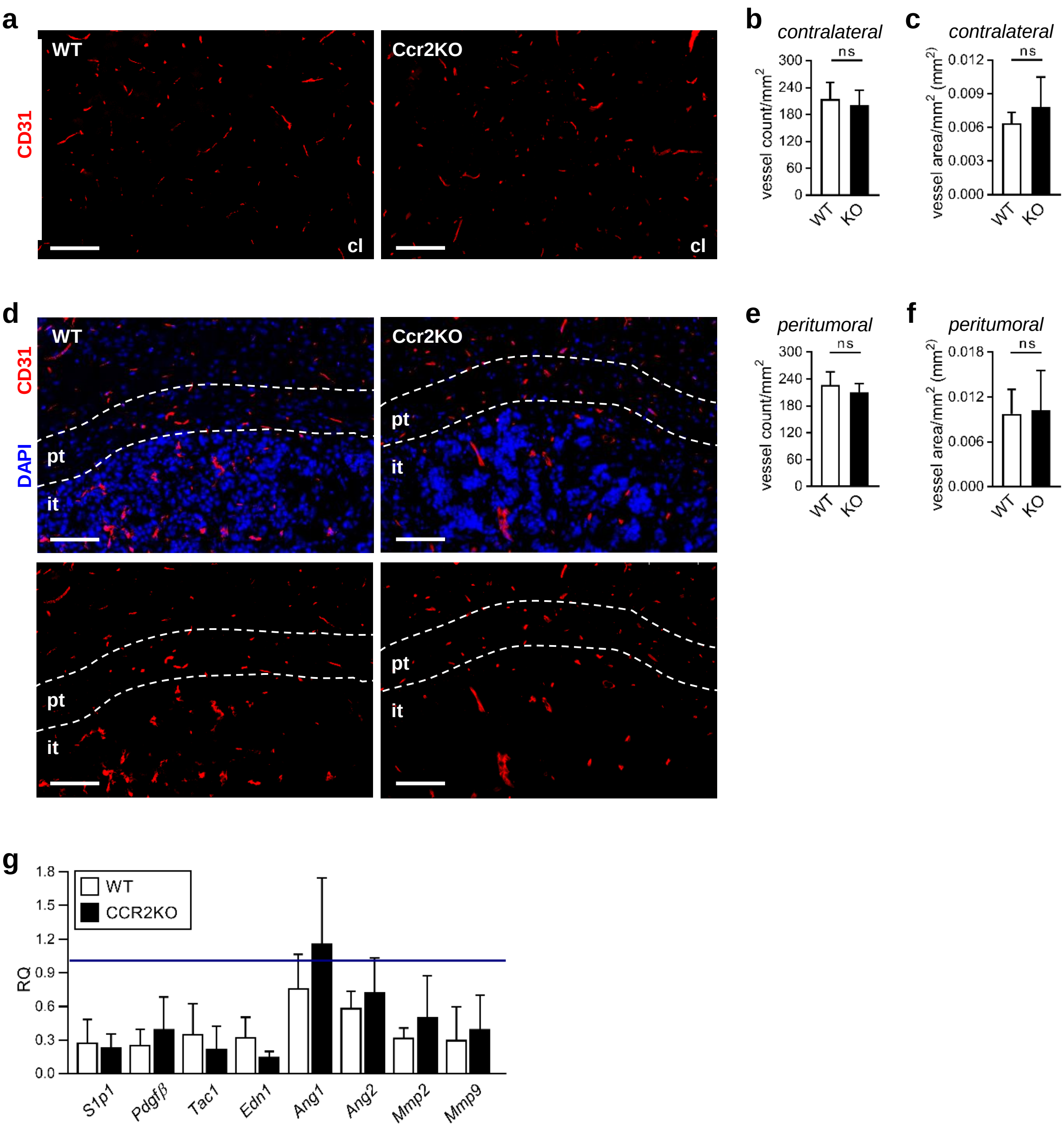

Figure S5. Vascular structure of brains is similar between mouse strains. **(a,d)** Representative images of immunofluorescence staining for CD31 of contralateral (cl) hemispheres **(a)** and the tumor border **(d)** of WT and Ccr2KO mouse brains. *Dashed lines* define peritumoral area (pt; it, intratumoral area). *Scale bars* 100 µm. **(b,c,e,f)** Graphs depict vessel numbers **(b,e)** and vascularized areas **(c,f)** of non-tumor bearing hemispheres (n= 5-6) **(b,c)** and peritumoral region (n= 6) **(e,f)**. ns, not significant (unpaired Student's t test). **(g)** RT-PCRs for indicated genes of CD11b$^+$ cells from tumor-bearing brain tissues of WT as well as Ccr2KO mice are presented (d21; n= 3-4). *Blue line* defines basic molecule expression in naïve WT mice.
