# Supplementary material for: CCR2 of Tumor Microenvironmental Cells Is a Relevant Modulator of Glioma Biology
Source: Cancers (Basel). 2020 Jul 13;12(7):1882. doi: 10.3390/cancers12071882 (PMC7408933; doi:10.3390/cancers12071882)
Supplement: Supplementary file 1 [file cancers-12-01882-s001.zip › MF Supplementary Tables.pdf]

CCR2 of Tumor Microenvironmental Cells is a Relevant Modulator of Glioma Biology

Matthäus Felsenstein, Anne Blank, Alexander D. Bungert, Annett Mueller, Adnan Ghori, Irina Kremenetskaia, Olga Rung, Thomas Broggini, Kati Turkowski, Lea Scherschinski, Jonas Raggatz, Peter Vajkoczy and Susan Brandenburg

Supplementary Tables

Table S1. Primer sequences.

gene	primer forward (5′-3′)	primer reverse (5′-3′)	reference
m18S	AACCCGTTGAACCCCATT	CCATCCAATCGGTAGTAGCG	[1]
mCcr2	GTTACCTCAGTTCATCCA	CAAGGCTCACCATCATCGTAGTC	[1]
mStat3	GAAGGGAGGCAAAGGGGAACA	ACTTGTCTAACAACCAACCCCC	designed
mArg1	AACACGGCAGTGGCTTTAACC	GGTTTTCATGTGGCGCATTC	[2]
mc-Myc	CACCAGCAGCGACTCTGAA	GCCCGACTCCGACCTCTTG	[3]
mYkl40	ATGCACACCTCTACTGAAGCC	ACCAGCTTGTACGCAGAGC	[4]
mIl10	CCCTGGGTGAGAAGCTGAAG	CACTGCCTTGCTCTTATTTTCACA	[5]
mTgfβ	TTGCTTCAGCTCCACAGAGA	TGGTTGTAGAGGGCAAGGAC	[5]
mStat1	CTGAATATTTCCCTCCTGGG	TCCCGTACAGATGTCCATGAT	[6]
mIrf7	CACCCCCATCTTCGACTTCA	CCAAAACCCAGGTAGATGGTGTA	[7]
mCox2	AGTTGATAACCGAGTCGTTCTG	CTGTTGCTTGATTTAGTC	[8]
mSmad7	GACTCCAGGACGCTGTTGGT	CCATGGTTGCTGCATGAACT	[9]
miNos	CGAAACGCTTCACTTCCAA	TGAGCCTATATTGCTGTGGCT	[2]
mTnfα	CACAGCCTTCCTCACAGAGC	GGAGGCAACAAGGTAGAGAGG	[5]
mIfnβ	CCAGCTCCAAGAAAGGACGA	CGCCCTGTAGGTGAGGTTGAT	[10]
mIl1α	GGCTCACTTCATGAGACTTGC	AGGTGTAAGGTGCTGATCTGG	[5]
mIl1β	ATCACTCATTGTGGCTGTGG	CATCTCGGAGCCTGTAGTGC	[5]
mIl6	GACTGATGCTGGTGACAACC	TTCTGCAAGTGCATCATCG	[5]
mIl12α	TACTAGAGAGACTTCTTCCACAACA AGAG	TCTGGTACATCTTCAAGTCCTCATAG A	[5]
mVegf	GAAGAAGAGGCCTGGTAATGG	AAGCCACTCACACACACAGC	[1]
mS1p1	TCAGGGAACTTTGCGAGTG	AGTGAGCCTTCAGTTACAGC	[11]
mPdgfβ	ATCCGCTCCTTTGATGATCT	GAGCTTTCCAACTCGACTCC	[12]
mTac1	GGCCAAGGAGAGCAAAGA	CGAGGATTTTCATGTTCGATT	[13]
mEdn1	GCACAACCGAGCACATTG	CCAGCCAGCATGGAGAGT	designed
mAng1	GCCTACACTTTCATTCTTCCAGA	TCTTCCTTGTGTTTTCCTTCCAT	[14]
mAng2	GGCAGCGTTGATTTTCAGAGGACT	TTTAATGCCGTTGAACTTATTTGT	[14]
mMmp2	CAGGGAATGAGTACTGGGTCTATT	ACTCCAGTTAAAGGCAGCATCTAC	[15]
mMmp9	ATCTCTTCTAGAGACTGGGAAGGAG	AGCTGATTGACTAAAGTAGCTGGA	[15]
h18S	GGCCCTGTAATTGGAATGAGTC	CCAAGATCCAACTACGAGCTT	[16]
hCCR2	TGTCCACATCTCGTTCTCGGT	CCGCTCTCGTTGGTATTTCTGA	[17]

Table S2. Basic expression of molecules.

gene	WT N (RQ ± SD)	Ccr2KO N (RQ ± SD)
Stat3	1.02 ± 0.24	1.30 ± 0.18
Arg1	1.02 ± 0.28	1.77 ± 1.90
c-Myc	1.03 ± 0.29	**0.54 ± 0.02**
Ykl40	1.12 ± 0.57	1.93 ± 1.01
Il10	1.02 ± 0.26	0.71 ± 0.20
Tgfβ	1.00 ± 0.09	**0.35 ± 0.13**
Stat1	1.08 ± 0.55	0.88 ± 0.29

Irf7	1.03 ± 0.33	**1.96 ± 0.48**
Cox2	1.01 ± 0.15	**6.29 ± 1.95**
Smad7	1.09 ± 0.52	**0.19 ± 0.07**
iNos	1.13 ± 0.66	0.71 ± 0.80
Tnfα	1.01 ± 0.18	0.83 ± 0.29
Ifnβ	1.05 ± 0.38	1.14 ± 0.20
Il1α	1.08 ± 0.49	0.43 ± 0.05
Il1β	1.04 ± 0.34	0.87 ± 0.42
Il6	1.03 ± 0.31	**0.36 ± 0.09**
Il12α	1.12 ± 0.65	**12.7 ± 5.83**

[bold]significant up- or down-regulated to WT N.

Reference

1. Brandenburg, S.; Muller, A.; Turkowski, K.; Radev, Y.T.; Rot, S.; Schmidt, C.; Bungert, A.D.; Acker, G.; Schorr, A.; Hippe, A.; et al. Resident microglia rather than peripheral macrophages promote vascularization in brain tumors and are source of alternative pro-angiogenic factors. *Acta Neuropathol.* **2016**, *131*, 365–378. DOI: 10.1007/s00401-015-1529-6
2. Nasser, M.W.; Qamri, Z.; Deol, Y.S.; Ravi, J.; Powell, C.A.; Trikha, P.; Schwendener, R.A.; Bai, X.F.; Shilo, K.; Zou, X.; et al. S100A7 enhances mammary tumorigenesis through upregulation of inflammatory pathways. *Cancer Res.* **2012**, *72*, 604–615. DOI: 10.1158/0008-5472.CAN-11-0669
3. Levy, J.; Cacheux, W.; Bara, M.A.; L'Hermitte, A.; Lepage, P.; Fraudeau, M.; Trentesaux, C.; Lemarchand, J.; Durand, A.; Crain, A.M.; et al. Intestinal inhibition of Atg7 prevents tumour initiation through a microbiome-influenced immune response and suppresses tumour growth. *Nat. Cell Biol.* **2015**, *17*, 1062–1073. DOI: 10.1038/ncb3206
4. Chung, C.; Kim, T.; Kim, M.; Kim, M.; Song, H.; Kim, T.S.; Seo, E.; Lee, S.H.; Kim, H.; Kim, S.K.; et al. Hippo-Foxa2 signaling pathway plays a role in peripheral lung maturation and surfactant homeostasis. *Proc. Natl. Acad. Sci. U. S. A.* **2013**, *110*, 7732–7737. DOI: 10.1073/pnas.1220603110
5. Brandenburg, S.; Turkowski, K.; Mueller, A.; Radev, Y.T.; Seidlitz, S.; Vajkoczy, P. Myeloid cells expressing high level of CD45 are associated with a distinct activated phenotype in glioma. *Immunol. Res.* **2017**, *65*, 757–768. DOI: 10.1007/s12026-017-8915-1
6. Shaul, M.E.; Bennett, G.; Strissel, K.J.; Greenberg, A.S.; Obin, M.S. Dynamic, M2-like remodeling phenotypes of CD11c+ adipose tissue macrophages during high-fat diet--induced obesity in mice. *Diabetes* **2010**, *59*, 1171–1181. DOI: 10.2337/db09-1402
7. Henault, J.; Martinez, J.; Riggs, J.M.; Tian, J.; Mehta, P.; Clarke, L.; Sasai, M.; Latz, E.; Brinkmann, M.M.; Iwasaki, A.; et al. Noncanonical autophagy is required for type I interferon secretion in response to DNA-immune complexes. *Immunity* **2012**, *37*, 986–997. DOI: 10.1016/j.immuni.2012.09.014
8. Lv, Y.J.; Yang, Y.; Sui, B.D.; Hu, C.H.; Zhao, P.; Liao, L.; Chen, J.; Zhang, L.Q.; Yang, T.T.; Zhang, S.F.; et al. Resveratrol counteracts bone loss via mitofilin-mediated osteogenic improvement of mesenchymal stem cells in senescence-accelerated mice. *Theranostics* **2018**, *8*, 2387–2406. DOI: 10.7150/thno.23620
9. Saika, S.; Ikeda, K.; Yamanaka, O.; Miyamoto, T.; Ohnishi, Y.; Sato, M.; Muragaki, Y.; Ooshima, A.; Nakajima, Y.; Kao, W.W.; et al. Expression of Smad7 in mouse eyes accelerates healing of corneal tissue after exposure to alkali. *Am. J. Pathol.* **2005**, *166*, 1405–1418. DOI: 10.1016/S0002-9440(10)62358-9
10. Watanabe, A.; Tatematsu, M.; Saeki, K.; Shibata, S.; Shime, H.; Yoshimura, A.; Obuse, C.; Seya, T.; Matsumoto, M. Raftlin is involved in the nucleocapture complex to induce poly(I:C)-mediated TLR3 activation. *J. Biol. Chem.* **2011**, *286*, 10702–10711. DOI: 10.1074/jbc.M110.185793
11. Liu, G.; Bi, Y.; Wang, R.; Yang, H.; Zhang, Y.; Wang, X.; Liu, H.; Lu, Y.; Zhang, Z.; Chen, W.; et al. Targeting S1P1 receptor protects against murine immunological hepatic injury through myeloid-derived suppressor cells. *J. Immunol.* **2014**, *192*, 3068–3079. DOI: 10.4049/jimmunol.1301193
12. Pozniak, K.N.; Pearen, M.A.; Pereira, T.N.; Kramer, C.S.M.; Kalita-De Croft, P.; Nawaratna, S.K.; Fernandez-Rojo, M.A.; Gobert, G.N.; Tirnitz-Parker, J.E.E.; Olynyk, J.K.; et al. Taurocholate Induces Biliary Differentiation of Liver Progenitor Cells Causing Hepatic Stellate Cell Chemotaxis in the Ductular Reaction: Role in Pediatric Cystic Fibrosis Liver Disease. *Am. J. Pathol.* **2017**, *187*, 2744–2757. DOI: 10.1016/j.ajpath.2017.08.024

13. Shi, C.; Das, V.; Li, X.; Kc, R.; Qiu, S.; I, O.S.; Ripper, R.L.; Kroin, J.S.; Mwale, F.; Wallace, A.A.; et al. Development of an in vivo mouse model of discogenic low back pain. *J. Cell. Physiol.* **2018**, *233*, 6589–6602. DOI: 10.1002/jcp.26280
14. El-Nabarawy, E.A.; El-Hanafy, G.M.; Rashed, L.A.; Yasin, F.S. Expression of angiopoietin-1, angiopoietin-2, and their receptor Tie2 in verruca vulgaris (common skin warts). *Int. J. Dermatol.* **2016**, *55*, e327–e331. DOI: 10.1111/ijd.13191
15. Sharma-Walia, N.; Patel, K.; Chandran, K.; Marginean, A.; Bottero, V.; Kerur, N.; Paul, A.G. COX-2/PGE2: molecular ambassadors of Kaposi's sarcoma-associated herpes virus oncoprotein-v-FLIP. *Oncogenesis* **2012**, *1*, e5. DOI: 10.1038/oncsis.2012.5
16. Kiselak, E.A.; Shen, X.; Song, J.; Gude, D.R.; Wang, J.; Brody, S.L.; Strauss, J.F., 3rd; Zhang, Z. Transcriptional regulation of an axonemal central apparatus gene, sperm-associated antigen 6, by a SRY-related high mobility group transcription factor, S-SOX5. *J. Biol. Chem.* **2010**, *285*, 30496–30505. DOI: 10.1074/jbc.M110.121590
17. Jakob, M.; Hemeda, H.; Janeschik, S.; Bootz, F.; Rotter, N.; Lang, S.; Brandau, S. Human nasal mucosa contains tissue-resident immunologically responsive mesenchymal stromal cells. *Stem Cells Dev* **2010**, *19*, 635–644. DOI: 10.1089/scd.2009.0245
